# Redetermination of 2,4′-methyl­ene­diphenol

**DOI:** 10.1107/S1600536811044989

**Published:** 2011-11-02

**Authors:** Wei-Dong Peng, Sheng-Chun Chen, Jie An, Fu-An Sun, Qun Chen

**Affiliations:** a Key Laboratory of Fine Petrochemical Technology, Changzhou University, Changzhou 213164, People’s Republic of China

## Abstract

In the previous determination [Finn & Musti (1950 ▶). *J. Soc. Chem. Ind. (London)*, **69**, S849] of the title compound, C_13_H_12_O_2_, the three-dimensional coordinates and displacement parameters were not reported. This redetermination at room temperature reveals that the dihedral angle between the benzene rings is 79.73 (6)°. In the crystal, inter­molecular O—H⋯O hydrogen bonds between adjacent mol­ecules result in two-dimensional wave-like supra­molecular motifs parallel to the *ab* plane.

## Related literature

For the previous determination, see: Finn & Musti (1950 ▶). For the importance of bis­phenol in industry, see: Patel & Patel (2009 ▶). For standard bond lengths, see: Allen *et al.* (1987 ▶).
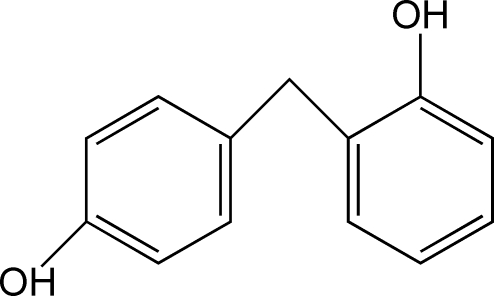


## Experimental

### 

#### Crystal data

C_13_H_12_O_2_
*M* *_r_* = 200.23
Monoclinic, 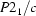

*a* = 5.0923 (5) Å
*b* = 15.3743 (14) Å
*c* = 13.2321 (12) Å
β = 96.660 (2)°
*V* = 1028.96 (17) Å^3^
*Z* = 4
Mo *K*α radiation
μ = 0.09 mm^−1^
*T* = 296 K
0.30 × 0.28 × 0.26 mm

#### Data collection

Bruker APEXII CCD area-detector diffractometer
Absorption correction: multi-scan (*SADABS*; Bruker, 2007 ▶) *T*_min_ = 0.975, *T*_max_ = 0.978
5900 measured reflections
1904 independent reflections
1404 reflections with *I* > 2σ(*I*)
*R*_int_ = 0.025

#### Refinement

*R*[*F* ^2^ > 2σ(*F* ^2^)] = 0.043
*wR*(*F* ^2^) = 0.144
*S* = 1.04
1904 reflections
138 parameters
H-atom parameters constrained
Δρ_max_ = 0.16 e Å^−3^
Δρ_min_ = −0.20 e Å^−3^

### 

Data collection: *APEX2* (Bruker, 2007 ▶); cell refinement: *APEX2* and *SAINT* (Bruker, 2007 ▶); data reduction: *SAINT*; program(s) used to solve structure: *SHELXTL* (Sheldrick, 2008 ▶); program(s) used to refine structure: *SHELXTL*; molecular graphics: *SHELXTL* and *DIAMOND* (Brandenburg, 2005 ▶); software used to prepare material for publication: *SHELXTL*.

## Supplementary Material

Crystal structure: contains datablock(s) I, global. DOI: 10.1107/S1600536811044989/zq2130sup1.cif

Structure factors: contains datablock(s) I. DOI: 10.1107/S1600536811044989/zq2130Isup2.hkl

Supplementary material file. DOI: 10.1107/S1600536811044989/zq2130Isup3.cml

Additional supplementary materials:  crystallographic information; 3D view; checkCIF report

## Figures and Tables

**Table 1:** Hydrogen-bond geometry (Å, °)

*D*—H⋯*A*	*D*—H	H⋯*A*	*D*⋯*A*	*D*—H⋯*A*
O1—H1⋯O2^i^	0.82	2.04	2.859 (2)	175
O2—H2*A*⋯O1^ii^	0.82	2.00	2.811 (2)	173

Symmetry codes: (i) 
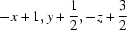
; (ii) 
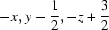
.

**Fig. 1.**
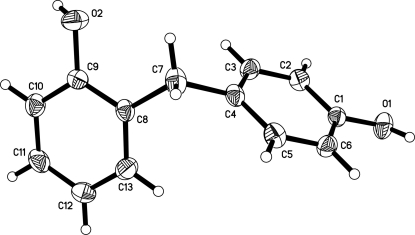
The molecular structure of the title compound with the atom-numbering scheme. Displacement ellipsoids are drawn at the 30% probability level.

**Fig. 2.**
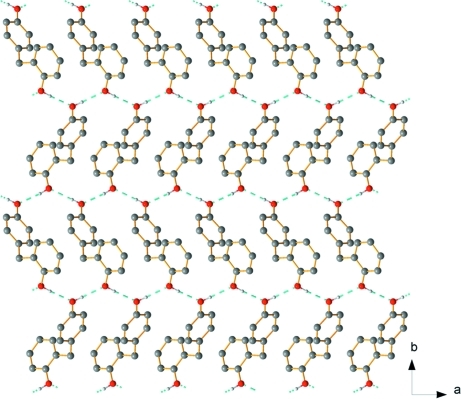
The two-dimensional structure of the title compound. Hydrogen atoms are omitted for clarity.

## supplementary crystallographic information

### Comment

2,4'-Dihydroxydiphenylmethane is one isomer of bisphenol F which is an important chemical and/or intermediate for the preparation of useful epoxy resins, phenolic resins, and polycarbonates in the plastic and rubber industries (Patel & Patel, 2009). This structure has been solved previously but with no available three-dimensional coordinates (Finn & Musti, 1950; CSD refcode: ZZZGWU). Herein, we present a redetermination at room temperature of the crystal structure of the title compound (Fig. 1). The bond lengths (Allen *et al.*, 1987) and angles are within normal ranges. The dihedral angle between the benzene rings is 79.73 (6)°. Intermolecular O—H···O hydrogen bonds between adjacent molecules result in two-dimensional wave-like supramolecular motifs along the *ab* plane (Fig. 2).

### Experimental

A 37% aqueous formaldehyde (20.31 g, 0.25 mol) solution was added to phenol (47.05 g, 0.50 mol) and oxalic acid (0.18 g, 1.40 mmol) at 70 °C with stirring for 4 h. Then the reaction mixture was condensed by vacuum distillation, affording a mixture of 4,4'-methylenebisphenol, 2,4'-methylenebisphenol and 2,2'-methylenebisphenol. By dissolving the resulting mixture (0.50 g) in the mixed solution of 2-propanol (20.0 ml) and water (10.0 ml), the needle colourless single crystals suitable for X-ray analysis were obtained after a slow evaporation of the solvents at room temperature for a period of about two weeks.

### Refinement

All H atoms bound to C atoms were assigned to calculated positions, with C—H = 0.97 Å (methylene) and 0.93 Å (aromatic), and refined using a riding model, with *U*_iso_(H) = 1.2*U*_eq_(C). The H atoms of the hydroxyl groups were firstly located in a difference Fourier map and then refined with the distance restraint O—H = 0.820 (1) Å, and finally constrained to ride on the O atom with *U*_iso_(H) = 1.5*U*_eq_(O).

### Crystal data

**Table:**

C_13_H_12_O_2_	*F*(000) = 424
*M**_r_* = 200.23	*D*_x_ = 1.293 Mg m^−^^3^
Monoclinic, *P*2_1_/*c*	Mo *K*α radiation, λ = 0.71073 Å
Hall symbol: -P 2ybc	Cell parameters from 1692 reflections
*a* = 5.0923 (5) Å	θ = 3.1–24.9°
*b* = 15.3743 (14) Å	µ = 0.09 mm^−^^1^
*c* = 13.2321 (12) Å	*T* = 296 K
β = 96.660 (2)°	Block, colourless
*V* = 1028.96 (17) Å^3^	0.30 × 0.28 × 0.26 mm
*Z* = 4	

### Data collection

**Table:**

Bruker APEXII CCD area-detector diffractometer	1904 independent reflections
Radiation source: fine-focus sealed tube	1404 reflections with *I* > 2σ(*I*)
graphite	*R*_int_ = 0.025
phi and ω scans	θ_max_ = 25.5°, θ_min_ = 2.0°
Absorption correction: multi-scan (*SADABS*; Bruker, 2007)	*h* = −6→6
*T*_min_ = 0.975, *T*_max_ = 0.978	*k* = −16→18
5900 measured reflections	*l* = −13→16

### Refinement

**Table:**

Refinement on *F*^2^	Primary atom site location: structure-invariant direct methods
Least-squares matrix: full	Secondary atom site location: difference Fourier map
*R*[*F*^2^ > 2σ(*F*^2^)] = 0.043	Hydrogen site location: inferred from neighbouring sites
*wR*(*F*^2^) = 0.144	H-atom parameters constrained
*S* = 1.04	*w* = 1/[σ^2^(*F*_o_^2^) + (0.0749*P*)^2^ + 0.2818*P*] where *P* = (*F*_o_^2^ + 2*F*_c_^2^)/3
1904 reflections	(Δ/σ)_max_ < 0.001
138 parameters	Δρ_max_ = 0.16 e Å^−^^3^
0 restraints	Δρ_min_ = −0.20 e Å^−^^3^

### Special details

**Table:**

Geometry. All e.s.d.'s (except the e.s.d. in the dihedral angle between two l.s. planes) are estimated using the full covariance matrix. The cell e.s.d.'s are taken into account individually in the estimation of e.s.d.'s in distances, angles and torsion angles; correlations between e.s.d.'s in cell parameters are only used when they are defined by crystal symmetry. An approximate (isotropic) treatment of cell e.s.d.'s is used for estimating e.s.d.'s involving l.s. planes.
Refinement. Refinement of *F*^2^ against ALL reflections. The weighted *R*-factor *wR* and goodness of fit *S* are based on *F*^2^, conventional *R*-factors *R* are based on *F*, with *F* set to zero for negative *F*^2^. The threshold expression of *F*^2^ > σ(*F*^2^) is used only for calculating *R*-factors(gt) *etc*. and is not relevant to the choice of reflections for refinement. *R*-factors based on *F*^2^ are statistically about twice as large as those based on *F*, and *R*- factors based on ALL data will be even larger.

### Fractional atomic coordinates and isotropic or equivalent isotropic displacement parameters (Å^2^)

**Table:**

	*x*	*y*	*z*	*U*_iso_*/*U*_eq_	
C1	0.4293 (4)	1.00937 (12)	0.83972 (14)	0.0379 (5)	
C2	0.2466 (4)	0.94424 (14)	0.84369 (15)	0.0441 (5)	
H2	0.1297	0.9454	0.8927	0.053*	
C3	0.2377 (4)	0.87678 (13)	0.77407 (15)	0.0441 (5)	
H3	0.1138	0.8327	0.7769	0.053*	
C4	0.4087 (4)	0.87356 (12)	0.70047 (14)	0.0393 (5)	
C5	0.5900 (4)	0.94019 (14)	0.69828 (15)	0.0455 (5)	
H5	0.7077	0.9392	0.6495	0.055*	
C6	0.6006 (4)	1.00814 (13)	0.76676 (15)	0.0438 (5)	
H6	0.7227	1.0528	0.7636	0.053*	
C7	0.3999 (4)	0.79840 (14)	0.62684 (17)	0.0548 (6)	
H7A	0.3855	0.7447	0.6644	0.066*	
H7B	0.5656	0.7966	0.5974	0.066*	
C8	0.1742 (4)	0.80219 (12)	0.54106 (14)	0.0384 (5)	
C9	0.0121 (4)	0.73108 (12)	0.51658 (14)	0.0381 (5)	
C10	−0.1909 (4)	0.73465 (14)	0.43729 (15)	0.0469 (5)	
H10	−0.2967	0.6861	0.4214	0.056*	
C11	−0.2353 (4)	0.81017 (15)	0.38226 (16)	0.0538 (6)	
H11	−0.3720	0.8128	0.3292	0.065*	
C12	−0.0785 (5)	0.88160 (15)	0.40538 (16)	0.0563 (6)	
H12	−0.1087	0.9327	0.3682	0.068*	
C13	0.1238 (4)	0.87742 (14)	0.48385 (17)	0.0514 (6)	
H13	0.2294	0.9261	0.4990	0.062*	
O1	0.4294 (3)	1.07617 (10)	0.90993 (12)	0.0556 (4)	
H1	0.5765	1.0985	0.9185	0.083*	
O2	0.0601 (3)	0.65582 (10)	0.57267 (12)	0.0551 (4)	
H2A	−0.0787	0.6291	0.5742	0.083*	

### Atomic displacement parameters (Å^2^)

**Table:**

	*U*^11^	*U*^22^	*U*^33^	*U*^12^	*U*^13^	*U*^23^
C1	0.0381 (10)	0.0335 (10)	0.0397 (10)	0.0055 (8)	−0.0058 (8)	−0.0021 (8)
C2	0.0392 (10)	0.0521 (12)	0.0416 (11)	−0.0007 (9)	0.0075 (8)	−0.0011 (9)
C3	0.0379 (11)	0.0413 (11)	0.0516 (12)	−0.0055 (8)	−0.0012 (9)	0.0010 (9)
C4	0.0345 (10)	0.0409 (11)	0.0396 (10)	0.0086 (8)	−0.0083 (8)	−0.0030 (8)
C5	0.0349 (10)	0.0595 (13)	0.0421 (11)	0.0011 (9)	0.0046 (8)	−0.0029 (9)
C6	0.0379 (10)	0.0425 (11)	0.0498 (12)	−0.0069 (8)	0.0008 (9)	0.0003 (9)
C7	0.0519 (13)	0.0518 (13)	0.0559 (13)	0.0186 (10)	−0.0146 (10)	−0.0147 (10)
C8	0.0382 (10)	0.0401 (11)	0.0358 (10)	0.0070 (8)	−0.0005 (8)	−0.0064 (8)
C9	0.0383 (10)	0.0390 (11)	0.0376 (10)	0.0056 (8)	0.0071 (8)	0.0017 (8)
C10	0.0421 (11)	0.0485 (13)	0.0480 (12)	−0.0038 (9)	−0.0031 (9)	−0.0053 (9)
C11	0.0525 (13)	0.0639 (15)	0.0409 (11)	0.0062 (11)	−0.0118 (9)	0.0020 (10)
C12	0.0654 (15)	0.0503 (14)	0.0503 (13)	0.0064 (11)	−0.0054 (11)	0.0125 (10)
C13	0.0530 (13)	0.0403 (12)	0.0584 (13)	−0.0035 (10)	−0.0038 (10)	0.0025 (10)
O1	0.0550 (9)	0.0489 (9)	0.0616 (10)	0.0010 (7)	0.0004 (8)	−0.0201 (7)
O2	0.0504 (9)	0.0460 (9)	0.0671 (10)	−0.0005 (7)	−0.0008 (7)	0.0169 (7)

### Geometric parameters (Å, °)

**Table:**

C1—C2	1.372 (3)	C7—H7B	0.9700
C1—C6	1.374 (3)	C8—C9	1.386 (3)
C1—O1	1.385 (2)	C8—C13	1.390 (3)
C2—C3	1.384 (3)	C9—O2	1.381 (2)
C2—H2	0.9300	C9—C10	1.386 (3)
C3—C4	1.381 (3)	C10—C11	1.375 (3)
C3—H3	0.9300	C10—H10	0.9300
C4—C5	1.382 (3)	C11—C12	1.371 (3)
C4—C7	1.509 (3)	C11—H11	0.9300
C5—C6	1.380 (3)	C12—C13	1.377 (3)
C5—H5	0.9300	C12—H12	0.9300
C6—H6	0.9300	C13—H13	0.9300
C7—C8	1.520 (3)	O1—H1	0.8200
C7—H7A	0.9700	O2—H2A	0.8200
			
C2—C1—C6	120.35 (18)	C8—C7—H7B	108.6
C2—C1—O1	117.60 (18)	H7A—C7—H7B	107.6
C6—C1—O1	122.04 (18)	C9—C8—C13	117.51 (17)
C1—C2—C3	119.42 (19)	C9—C8—C7	121.52 (18)
C1—C2—H2	120.3	C13—C8—C7	120.97 (18)
C3—C2—H2	120.3	O2—C9—C8	118.16 (17)
C4—C3—C2	121.45 (19)	O2—C9—C10	120.67 (18)
C4—C3—H3	119.3	C8—C9—C10	121.17 (18)
C2—C3—H3	119.3	C11—C10—C9	119.79 (19)
C3—C4—C5	117.77 (18)	C11—C10—H10	120.1
C3—C4—C7	120.60 (19)	C9—C10—H10	120.1
C5—C4—C7	121.62 (19)	C12—C11—C10	120.15 (19)
C6—C5—C4	121.51 (19)	C12—C11—H11	119.9
C6—C5—H5	119.2	C10—C11—H11	119.9
C4—C5—H5	119.2	C11—C12—C13	119.8 (2)
C1—C6—C5	119.49 (19)	C11—C12—H12	120.1
C1—C6—H6	120.3	C13—C12—H12	120.1
C5—C6—H6	120.3	C12—C13—C8	121.6 (2)
C4—C7—C8	114.59 (16)	C12—C13—H13	119.2
C4—C7—H7A	108.6	C8—C13—H13	119.2
C8—C7—H7A	108.6	C1—O1—H1	109.5
C4—C7—H7B	108.6	C9—O2—H2A	109.5
			
C6—C1—C2—C3	0.6 (3)	C4—C7—C8—C13	49.1 (3)
O1—C1—C2—C3	179.23 (16)	C13—C8—C9—O2	179.89 (18)
C1—C2—C3—C4	−0.1 (3)	C7—C8—C9—O2	0.2 (3)
C2—C3—C4—C5	−0.1 (3)	C13—C8—C9—C10	0.7 (3)
C2—C3—C4—C7	178.59 (17)	C7—C8—C9—C10	−179.05 (18)
C3—C4—C5—C6	−0.3 (3)	O2—C9—C10—C11	−179.92 (19)
C7—C4—C5—C6	−178.93 (18)	C8—C9—C10—C11	−0.7 (3)
C2—C1—C6—C5	−0.9 (3)	C9—C10—C11—C12	0.3 (3)
O1—C1—C6—C5	−179.51 (17)	C10—C11—C12—C13	0.1 (4)
C4—C5—C6—C1	0.8 (3)	C11—C12—C13—C8	−0.1 (4)
C3—C4—C7—C8	77.4 (3)	C9—C8—C13—C12	−0.3 (3)
C5—C4—C7—C8	−104.0 (2)	C7—C8—C13—C12	179.5 (2)
C4—C7—C8—C9	−131.1 (2)		

### Hydrogen-bond geometry (Å, °)

**Table:**

*D*—H···*A*	*D*—H	H···*A*	*D*···*A*	*D*—H···*A*
O1—H1···O2^i^	0.82	2.04	2.859 (2)	175
O2—H2A···O1^ii^	0.82	2.00	2.811 (2)	173

Symmetry codes: (i) −*x*+1, *y*+1/2, −*z*+3/2; (ii) −*x*, *y*−1/2, −*z*+3/2.
